# Transcriptomic analysis of zebrafish prion protein mutants supports conserved cross-species function of the cellular prion protein

**DOI:** 10.1080/19336896.2021.1924557

**Published:** 2021-06-18

**Authors:** Niall Mungo Pollock, Patricia Leighton, Gavin Neil, W. Ted Allison

**Affiliations:** a Department of Biological Sciences, University of Alberta, Edmonton, Canada; b Centre for Prions & Protein Folding Disease, University of Alberta, Edmonton, Canada; c Department of Medical Genetics, University of Alberta, Edmonton, Canada

**Keywords:** Prion knockout, transcriptome, RNA-sequencing, cell adhesion, scrapie

## Abstract

Cellular Prion Protein (PrP^C^) is a well-studied protein as the substrate for various progressive untreatable neurodegenerative diseases. Normal functions of PrP^C^ are poorly understood, though recent proteomic and transcriptomic approaches have begun to reveal common themes. We use our compound *prp1* and *prp2* knockout mutant zebrafish at three days post fertilization to take a transcriptomic approach to investigating potentially conserved PrP^C^ functions during development. Gene ontology analysis shows the biological processes with the largest changes in gene expression include redox processing, transport and cell adhesion. Within these categories several different gene families were prevalent including the solute carrier proteins, cytochrome p450 enzymes and protocadherins. Continuing from previous studies identifying cell adhesion as an important function of PrP^C^ we found that in addition to the protocadherins there was a significant reduction in transcript abundance of both *ncam1a* and *st8sia2*. These two genes are involved in the early development of vertebrates. The alterations in cell adhesion transcripts were consistent with past findings in zebrafish and mouse prion protein mutants; however E-cadherin processing after prion protein knockdown failed to reveal any differences compared with wild type in either our double *prp1/prp2* mutant fish or after *prp1* morpholino knockdown. Our data supports a cross species conserved role for PrP^C^ in the development and maintenance of the central nervous system, particularly by regulating various and important cell adhesion processes.

## Introduction

The cellular prion protein (PrP^C^) is a well-conserved protein across mammals and to a lesser extent across other vertebrates. It has fascinated researchers since its identification as the cause of a variety of neurodegenerative disorders including Creutzfeldt Jakob disease (CJD) in humans, scrapie in sheep, chronic wasting disease (CWD) in cervids and bovine spongiform encephalopathy (BSE) in cattle via a conformational change in PrP^C^ to become scrapie prion protein, or PrP^Sc^ [1,2]. Interest is often focussed on the infectious capabilities of the PrP^Sc^ conformation to spread disease, including across species, dubbed ‘the protein only hypothesis’[2]. In addition to its ability to misfold into PrP^Sc^, normally folded PrP^C^ has been implicated in the pathology of Alzheimer’s disease by acting as a receptor for soluble amyloid-beta oligomers [3–7]. Despite being subjected to such a large amount of scrutiny, the actual normal physiological functions of PrP^C^ are not well understood, nor how these functions may be affected under disease conditions [8]. Here we perform transcriptomic analysis on wild-type (WT) vs mutant zebrafish, which lack both *prp1* and *prp2* gene products to identify potential functions of PrP^C^ during early development.

**Figure 1.**
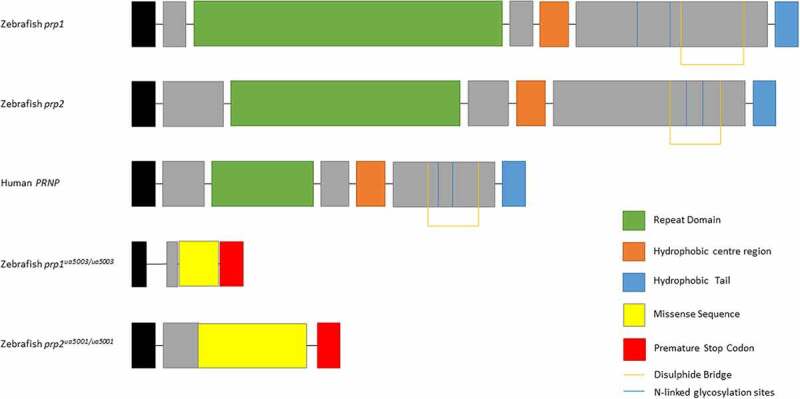
Prion proteins of zebrafish Prp1, Prp2, human PrP^C^ and Prp1*^ua5003/ua5003^* and Prp2*^ua5001/ua5001^* mutant proteins. Zebrafish prion protein genes are larger however have the same conserved domains as human *PRNP*: the repeat domain (green), hydrophobic centre region (orange), hydrophobic tail (blue), N-linked glycosylation sites (blue lines) and di-sulphide bridge (orange lines). The *prp1^ua5003/ua5003^* and *prp2^ua5001/ua5001^* alleles have frameshift deletions near the beginning of the coding exon, leading to a missense sequence of amino acids (yellow), pre-mature stop codons (red) and a shortened, nonsense transcript

Zebrafish possesses two prion protein genes homologous to mammalian *PRNP, prp1* and *prp2*, due to a whole genome duplication which occurred in the teleost lineage [9,10]. While both *prp1* and *prp2* are larger than their mammalian counterpart and therefore share little similarity at the amino acid level, all predicted functional domains of PrP^C^ are present in both including an N-terminal signal peptide, a repetitive region, a central hydrophobic domain, a disulphide bridge, two N-linked glycosylation sites and a GPI anchor for attachment to the cell membrane [11] (Figure 1). This conservation of PrP across evolutionary time indicates that this protein has ancient and important physiological functions.

Transmission of prion diseases to fish by crossing the species barrier has been previously investigated. The difference in size of mammalian to fish PrPs and lower conservation means the chance of transmissibility between species is low but the conserved domains may suggest it is not impossible. Studies have shown that sea bream fed with either scrapie or BSE contaminated brain homogenate results in signs of neurodegeneration and deposits in the brain which reacted to antibodies against sea bream PrP. These deposits developed faster in fish challenged with BSE prions and did not occur in those fed with non-contaminated brain homogenate [12]. While deposits and histological signs of neurodegeneration were observed, there were no clinical symptoms of prion disease and passaging the disease onto additional animals was not reported. Additional *in vitro* studies using mouse cell culture demonstrated that three different fish PrP proteins, including zebrafish Prp1 and Prp2, did not increase the formation of proteinase K resistant prion conversion [13]. While this supports that it is unlikely fish Prps can misfold into pathogenic species after exposure to mammalian prions, the various nature of different PrP^Sc^ strains means it still remains a possibility.

There have been many proposed functions for PrP^C^ including cell adhesion, learning and memory, maintaining circadian rhythm, aspects of the immune response, synaptic function, neuroprotection and more [14,15]. Determining which of these is a direct function of PrP^C^ has proven difficult since animal knockout studies have not shown any obvious overt phenotype in both mice and zebrafish models [16,17]. This is in stark contrast to what can be seen after acute knockdown of PrP^C^, such as morpholino knockdown of *prp1* in zebrafish leading to a lethal phenotype during gastrulation [18]. This phenotype is particularly interesting due to a similar phenotype occurring after knockdown of certain ZIP proteins, from which PrP^C^ may be phylogenetically linked [19]. Discrepancies between chronic stable knockout of PrP^C^ and acute knockdown may suggest robust compensatory mechanisms in mutants allowing for their survival. The lack of overt phenotypes after *Prnp* gene knockout is surprising as PrP^C^ is evolutionarily well conserved which would suggest an essential function; yet there is little evidence for any particular gene(s) which may be involved in functional redundancy.

Studies in zebrafish, from our own lab and others, support a conserved role of zebrafish prion proteins in cell adhesion [18,20]. In addition, proteomic analysis in cell culture has revealed a robust role for mammalian PrP^C^ during epithelial–mesenchymal transition, a cell adhesion event during development, through affecting NCAM1 polysialylation via ST8SIA2 production [21,22]. These studies suggest it plays an important role in the early development of vertebrates and possibly subsequently acts to maintain areas in which it is expressed. Therefore, we have carried out RNA-sequencing analysis on zebrafish larvae to further investigate the role of PrP^C^ during development.

## Results

### Compound homozygous prp1^ua5003;ua5003^; prp2^ua5001;ua5001^ knockout mutant exhibited transcriptomic changes

**Figure 2.**
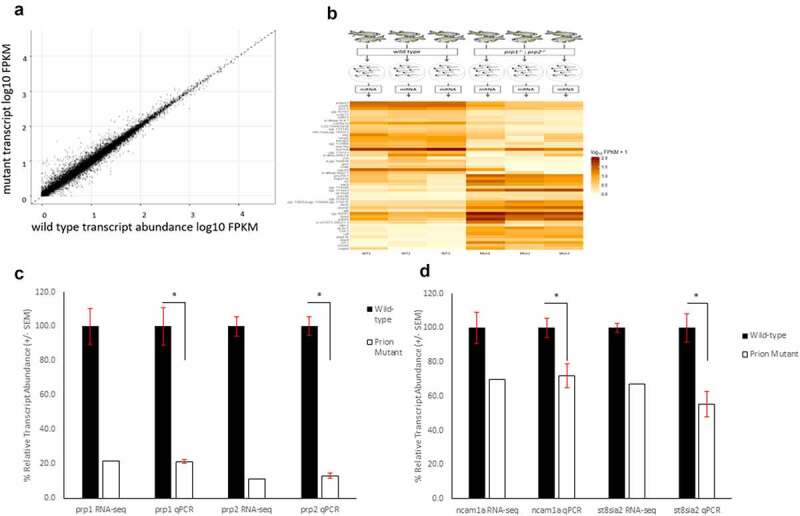
RNA-Sequencing show 1249 genes with an increase or decrease of log_2_ fold change of 0.5 between wild-type and compound homozygous prion mutant zebrafish larvae. (A) Scatter graph showing relative FPKM values for wild-type (X-axis) and mutant (Y-axis) genes after RNA-sequencing. (B) Methodology diagram showing RNA-sequencing workflow. Two groups, wild-type and prion mutant (*prp1^ua5003/ua5003^; prp2^ua5001/ua5001^*) homozygous fish, each with three replicates containing a pool of 50 3dpf larvae were processed and sent for RNA-sequencing. Heatmap displays sample of the 25 genes with a biggest differential abundance in FPKM in wild-types compared to mutants. (C) Relative transcript abundance between wild-type and prion mutant fish comparing *prp1* and *prp2* through both RNA-sequencing and RT-qPCR analysis. D) Relative transcript abundance of *ncam1a* and *st8sia2* through both RNA-sequencing and RT-qPCR analysis. * = P < 0.05

WT and *prp1^ua5003/ua5003^; prp2^ua5001/ua5001^* homozygous compound mutant zebrafish larvae underwent RNA-sequencing analysis. Prion compound mutant fish have engineered small deletion mutations near the beginning of the coding sequence leading to frameshifts in each gene, premature stop codons causing truncated proteins and predicted loss of function [17,23] (Figure 1). Three pools of 50 3dpf WT AB fish and *prp1^ua5003/ua5003^; prp2^ua5001/ua5001^* compound homozygous mutant fish were collected and sent to Otogenetics for RNA-sequencing (Figure 2). The age of 3dpf was chosen because it represents a time point, early in development of zebrafish, when the CNS is present, the embryo is available for genetic manipulation and where there is expected to be an overlap in the expression of both *prp1* and *prp2* [11]. Using a fold change cut-off of log_2_0.5 (i.e. there is either 50% more or 50% less transcript abundance) we found a significant change in the transcript abundance of 1249 genes, with 745 showing an increase in transcript abundance and 504 showing a decrease in transcript abundance in compound mutant *prp1^ua5003/ua5003^; prp2^ua5001/ua5001^* fish compared to WT (Figure 2a and Table 1). We have previously shown a decrease in relative transcript abundance of *prp1* and *prp2* in *prp1^ua5003/ua5003^; prp2^ua5001^* mutants, predicted to be due to nonsense mediated decay of nonsense mRNA [17], and as expected *prp1* and *prp2* were among the top genes showing a decrease in transcript abundance.

**Table 1.** Total number of genes with either a significant increase or decrease in transcript abundance between wild-type and *prp1^ua5003/ua5003^; prp2^ua5001/ua5001^* homozygous mutant fish with a log_2_ fold change equal to or greater than 0.5

Total number of genes with a log2 fold change of 0.5 or greater	1249
Increase in transcript abundance	745 (60%)
Top 10	cel.1, zp3a.2, zgc:173,443, c3a.3, c6ast4, dpp4, paqr3b, prss59.1, pde6h, ela3l
Decrease in transcript abundance	504 (40%)
Top 10	ghrh, zgc:194,878, capn2l, mhc1lia, krtcap2, col28a1a, irx4b, prnprs3, si:dkeyp-94g1.1, zgc:112,966

RT-qPCR experiments for both *prp1* and *prp2* confirmed a significant reduction in transcript abundance for both genes in our mutants of 79% and 87% respectively (Figure 2c). Initial RT-qPCR of select genes (implicated in eye development) does not strongly support validation of the RNA-sequencing results, this could be due the circadian nature of the expression of those genes, and variability due to the low transcript abundance perhaps being difficult to detect through RT-qPCR, though we have yet to prove either explanation (Supplementary Figure 1). On the other hand, changes in transcript abundance for several other genes were verifiable by RT-qPCR (Figure 2d and described below). For the full results, see the published transcriptome (GEO accession: GSE164423).

Amongst the ten genes showing the largest increase in transcript abundance in mutants compared to WT, five have been linked to proteolytic/hydrolytic processes (*cel.1, ela3l, prss59.1, dpp4* and *c6ast4*). While the proteolytic processing of PrP^C^ itself is becoming increasingly well documented [24–26], its actions in the proteolytic processing of other molecules, whether directly or indirectly is somewhat less appreciated. PrP^C^ is becoming increasingly associated with cell adhesion [18,27,28], proliferation [29,30] and signalling and it is possible it acts in complexes that process other proteins and molecules as part of these events.

Among the ten genes with the biggest decrease in transcript abundance there does not appear to be a consistent biological process linking them. The gene with the biggest reduction in relative transcript abundance is growth hormone releasing hormone (*ghrh*). Like in mammals Ghrh causes increases in the release of growth hormone during development, particularly in the central nervous system and gut. Secretion of Ghrh is controlled in a circadian manner and has antagonistic effects to somatostatin, with Ghrh promoting short wave sleep while somatostatin promotes deeper REM sleep [31]. Growth hormone levels decrease with age and have been suggested to be involved in the ageing process related to a decrease in physiological functions controlled by the hypothalamus [32]. Interestingly, both somatostatin 1 and somatostatin receptor 5 show a significant increase in transcript abundance in prion mutants (60% and 65% respectively). Through recent collaborations we have shown a disruption in the sleep/wake cycle of *prp1^ua5003/ua5003^; prp2^ua5001/ua5001^* mutant zebrafish after exposure to amyloid-beta oligomers [7].

### Gene ontology analysis of biological processes affected in prp1 and prp2 mutant zebrafish

**Figure 3.**
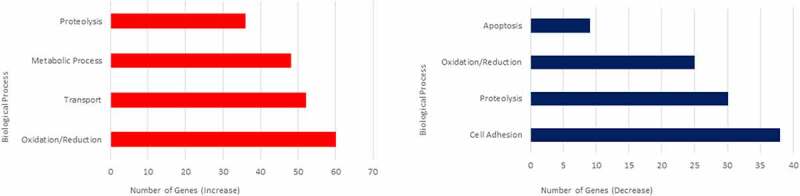
Biological Process Gene Ontologies most affected in 3dpf prion mutant (*prp1*^ua5003/ua5003^; *prp2^ua5001/ua5001^*) zebrafish compared to wild type. Biological processes showing genes with the greatest increase in transcript abundance are shown on the left (red), and genes with the biggest decrease in transcript abundance are shown on the right (blue)

The most populous Biological Process categories of genes altered in zebrafish prion mutants are reported in Figure 3. Amongst the processes exhibiting a significant increase in transcript abundance, the oxidation/reduction category is represented most often with genes showing a significant increase in transcript abundance (Table 2). Gene ontology analysis for genes with a significant decrease in transcript abundance again shows a similar trend to processes previously linked with PrP^C^ [33]. Table 3 shows the most populated biological process categories with a significant decrease in transcript abundance. Cell adhesion is the largest, with the majority of genes belonging to the protocadherin (*pcdh*) family showing a significant reduction in transcript abundance, totalling 31 out of the 38 cell adhesion genes. The *pcdh* genes affected belong to the *pcdh2* alpha and gamma sub clusters. Protocadherins are thought to be particularly involved in the cell adhesion of the early central nervous system [34], and this reduction in transcript abundance in our mutant fish may in the future help shed light on some of our previous findings suggesting a delay in neural development after prion protein knockdown [35].

**Table 2.** Most populated biological process gene ontologies for genes with a log_2_ fold change of 0.5 or greater

Category	Genes	Number of genes
Oxidation Reduction Process	hmgcra, haao, hpda, foxred2, nsdhl, aldh1l1, aldh7a1, aox6, cyp1a, cyp2aa1, cyp2aa4, cyp2aa6, cyp2ad2, cyp2ad3, cyp2k18, cyp2k16,	60
	cyp2n13, cyp2p8, cyp2p9, cyp2x7, cyp2x9, cyp2k19, cyp24a1, cyp27a1.4, cyp46a1.1, cyp51, cyp7a1, cyp8b1, cyb5r2, dio1, ero1a, fads2,	
	gcdhb, gmpr2, hsd3b7, kmo, ldhbb, msmo1, pipox, pcyox1, pdha1b, rpe65a, rdh8b, sdr42e1, si:dkey-180p18.9, si:dkey-91i10.3, sqlea,	
	srd5a2a, sc5d, sod1, tbxas1, tm7sf2, tdo2a, tyr, uox, zgc:101,765, zgc:110,783, zgc:136,333, zgc:66,484, zgc:77,938	
Transport	atp5j, abca4a, rhcgb, snf8, ap1m2, apoda.2, apodb, aqp8a.1, aqp8a.2, aqp9b, chmp1a, ero1a, fabp10a, fabp2, fads2, gabra6a, gabrr2a,	52
	gabrz, gc, hbbe2, hbz, mb, kcnc1b, kcnf1a, ptgdsb.1, ptgdsb.2, p2rx2, p2rx4a, rlbp1b, rbp2a, rbp2b, rbp4l, snupn, slc1a7b, slc15a1b,	
	slc20a1a, slc25a10, slc25a3a, slc5a1, slc5a11, slc51a, slc52a3, slc6a14, slc6a11a, slc6a13, slc6a19b, slco1d1, spns3, ttpa, tfa, trpv6,	
	zgc:153,704, mmp9, metap2a, nln, prep, si:dkey-269i1.4, tll1, tinagl1, usp20, zgc:112,285, zgc:174,153, zgc:174,855	
Metabolic Process	agpat2, hmgcs1, hoga1, aclya, ugt1ab, ugt1a1, ugt1a2, ugt1a4, ugt1a5, ugt1a6, ugt1a7, ugt2a1, ugt2a2, ugt2a3, ugt2a4, ugt2b1, ugt2b3,	48
	ugt2b5, ugt5b1, ugt5b3, ugt5b4, ugt5d1, ugt8, acaa1, aldh1l1, aldh7a1, alpi.2, alas1, fah, gla, gcdhb, gstm.3, gstm.1, gsto1, mettl7a, pmt,	
	pfkmb, pdha1b, si:ch211-93g23.2, slc27a1b, scp2a, tyr, uck1, zgc:101,040, zgc:101,540, zgc:101,569, zgc:162,780, zgc:66,313	
Proteolysis	Ihha, lonrf1l, anpepa, anpepb, ace2, cpa4, cpa5, cpb1, cpb2, caspb, ctsba, ctsl.1, ctsla, ctrl, ctrb1, cfd, ela2l, ela2, ela3l, furinb, enpep,	36
	irbp, pcsk1, prss59.1, prss59.2, prss60.2, si:dkey-194e6.1, c6ast4, try, zgc:100,868, zgc:112,160, zgc:112,302, zgc:136,872, zgc:85,932, zgc:92,041,	
	zgc:92,480	
Visual Perception	abca4a, grk7a, irbp, opn1lw2, opn1mw1, opn1sw1, opn1sw2, prph2a, prph2b, pde6h, rgra, rom1b, rpe65a, rlbp1b, rho, zgc:73,359	16

**Table 3.** Most populated biological process gene ontologies for genes with a log_2_ fold change of −0.5 or less

Category	Genes	Number of genes
Cell Adhesion	cdh5, cntn2, cyr61 l1, itga9, tln2a, tinagl1, si:ch211-66e2.3, pcdh2aa1, pcdh2aa15, pcdh2aa3, pcdh2ab1, pcdh2ab10, pcdh2ab11, pcdh2ab12,	38
	pcdh2ab3, pcdh2ab5, pcdh2ab6, pcdh2ab7, pcdh2ab8, pcdh2ab9, pcdh2ab2, pcdh2ac, pcdh2g1, pcdh2g10, pcdh2g12,	
	pcdh2g13, pcdh2g16, pcdh2g17, pcdh2g2, pcdh2g28, pcdh2g29, pcdh2g3, pcdh2g4, pcdh2g5, pcdh2g6, pcdh2g7, pcdh2g8, pcdh2g9	
Proteolysis	agtpbp1, cflara, ank2b, atg4c, capn12, capn2l, capn7, capn8, casp2, casp3b, casp6, casp6l1, ctsd, ctslb, f2rl1.2, f9b, f7i, he1b, mmp30,	30
	mmp9, metap2a, nln, prep, si:dkey-269i1.4, tll1, tinagl1, usp20, zgc:112,285, zgc:174,153, zgc:174,855	
Oxidation Reduction Process	dhcr7, dao.1, dao.2, sh3pxd2aa, aldh18a1, cyp2aa3, cyp2aa9, gpd1l, hmox1a, loxl1, loxl2b, mdh1ab, ogdha, p4ha2, p4ha1a, p4ha1b,	25
	ptgis, ptgr1, pyroxd2, pycr1b, si:dkey-239i20.4, suox, txnl1, ywhae2, cyp2aa2	
Regulation of Apoptosis	bag6l, cflara, dnaja3a, casp2, gdf11, mcl1b, pmaip1, prnprs3, ptgis	9

### Prion protein is involved in cell adhesion processes in early larval development

Previous work has established a link between PrP^C^ and cell adhesion. Schmitt-Ulms and colleagues used a proteomic and transcriptomic approach in PrP^C^ knockout cells, to show a role for PrP^C^ in the polysialylation of Ncam1 [21]. In zebrafish, Malaga-Trillo and colleagues found a link between Prp1 and cell adhesion including, though not necessarily limited to, effects on the maturation of E-cadherin during embryogenesis [18].

Results from our RNA-sequencing data do not show a significant difference in the transcript abundance of E-cadherin between mutants and WT, though this is not surprising if the role of prion protein is in the maturation of the protein (a proteolytic event) and not of the expression of the gene. In zebrafish, *ncam1a* is a homologue of NCAM1 and the Ncam1a protein is also polysialylated by St8sia2 [36]. There is a 30% reduction in the transcript abundance of *ncam1a* and a 33% reduction in the transcript abundance of *st8asia2* in our mutant fish compared to WT (Figure 2d). We confirmed this through RT-qPCR, finding a similar reduction in transcript abundance of *ncam1a*, of approximately 30%, and 50% for *st8asia2*.

**Figure 4.**
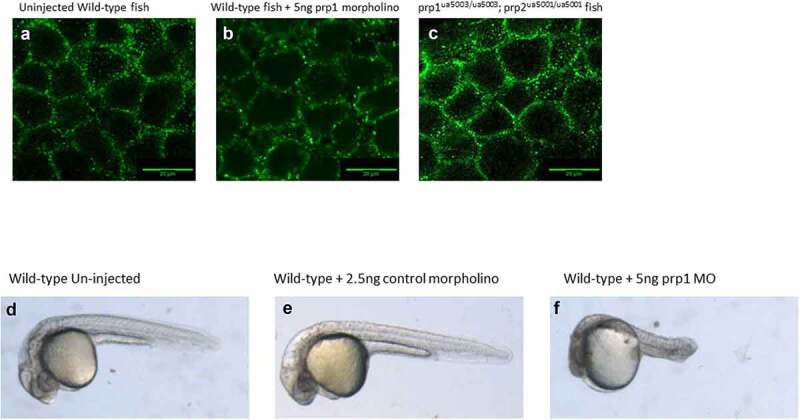
(A–C) There does not appear to be a difference in the maturation or localization of e-cadherin in either 5ng *prp1* morpholino injected AB zebrafish or uninjected *prp1^ua5003/ua5003^; prp2^ua5001/ua5001^* homozygous mutant fish compared to uninjected wild-type controls. (F–E) After 3dpf wild-type larvae injected with *prp1* MO (f) show significant signs of necrosis and developmental abnormalities compared to uninjected and control injected larvae (d, e)

After establishing these changes in *ncam1a* and *st8sia2* transcript abundance we next looked at whether there were changes in the processing of E-cadherin in our prion mutant fish compared to WT. Previous work has established a role of prp1 in regulating E-cadherin processing in zebrafish [18], and our lab has previously shown changes in both E-cadherin and β-catenin localization after morpholino knockdown of *prp2* [20]. Zebrafish embryos for both WT, compound mutant fish and *prp1* morpholino injected fish were stage-selected for those entering the shield stage of embryogenesis, approximately 6 hpf. We were not able to identify any changes to the processing or localization of E-cadherin either in our *prp1^ua5003/ua5003^; prp2^ua5001/ua5001^* mutant fish or WT fish injected with 5ng *prp1* morpholino (Figure 4(a-c)). We kept morpholino injected fish and control injected to fish to see if the morpholino was influencing the fish as they developed. We did not see a significant increase in the number of embryos perishing after 1dpf between the morpholino and control injected embryos (data not shown). By 3dpf morpholino injected fish showed clear signs of necrosis and developmental abnormalities compared to the control injected and un-injected control (Figure 4(d-f)). These results would suggest that morpholino knockdown of *prp1* was causing an effect compared to the control injected fish. Why this effect is different compared to what has been previously published is not immediately clear. Morpholinos have come under increased scrutiny due to differences seen in morphants compared to mutants; however this could be due to acute knockdown of genes having more impact than stable, chronic knockout [37,38]. We previously discussed at length potential explanations for the disparate results during acute knockdown vs. stable mutation of prion proteins [17] and concluded that results from these morpholino reagents should be interpreted with caution.

### KEGG analysis shows decreased transcript abundance in focal adhesion and actin cytoskeleton regulation pathways

The most affected pathway in prion mutants is metabolism, exhibiting both an increase and decrease in relative transcript abundance; however due to the sheer size of this KEGG pathway there was little consistency in processes affected; therefore we focussed our attention on the next most populous pathways.

**Figure 5.**
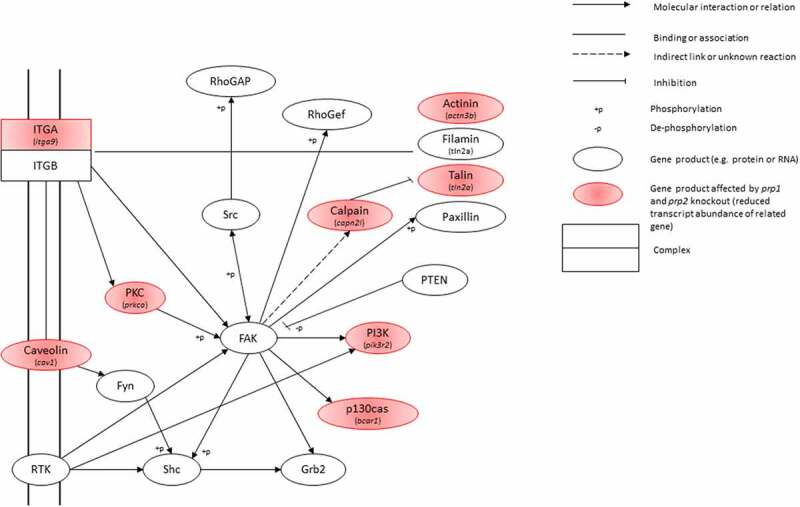
Snapshot of the Focal Adhesion Kinase KEGG pathway. Gene products highlighted in red show those with a significant decrease in transcript abundance, with the specific gene italicized underneath

KEGG analysis shows the two most populated pathways with genes having a decrease in transcript abundance are the focal adhesion kinase (FAK) pathway and actin cytoskeleton regulation pathway. There are two FAK homologues in zebrafish, *ptk2ab* (*fak1a*) and *ptk2aa* (*fak1b*). While neither shows a significant change in transcript abundance in our zebrafish mutants, the FAK pathway does show several genes with a significant reduction in transcript abundance in close proximity to the FAK genes in the pathway. Genes with direct interactions with FAK showing a significant decrease in transcript abundance include members of the calpain, actinin, talin and integrin families (Figure 5). There is a significant reduction in transcript abundance in *capn2l, tln2a, actn3b* and *bcar1*. All of these have been heavily linked with the regulation of the actin cytoskeleton, affecting cell mobility, division and differentiation [39–43]. There is considerable overlap between genes affected in the FAK pathway and the regulation of the actin cytoskeleton pathway:*raf1b, actn3b, bcar1, capn2l, itga9, pak6b, pik3r2, rac1b* show a significant decrease in transcript abundance in both.

Taken alongside the large number of protocadherin family members also showing a reduction in transcript abundance (Table 3), as well as *ncam1a* and *st8sia2* this suggests that *prp1* and *prp2* help regulate the processes of cell adhesion and differentiation during early development.

## Discussion:

### Conserved roles of PrP^C^ across species

Despite numerous animal knockout models, there is yet to be a clear and obvious phenotype attributed to the loss of PrP^C^. This could be due to the age at which the animals were being observed, with evidence both from our lab and others that PrP^C^ may be important in the early development of organisms. Acute transient knockdown of Shadoo (‘shadow of prion protein’) in PrP^C^ knockout mice led to embryonic lethality [44], though this effect was not seen in a combined knockout model of Shadoo and PrP^C^ [45]. Morpholino knockdown of *prp1* in zebrafish led to arrest during gastrulation attributed to deficits in cell adhesion [18,46] and our own analysis of *prp1* and *prp2* in zebrafish suggests further, if non-essential, roles in early development [17,20,35]. This does not account for the relatively diverse and high expression levels of prion protein after development and throughout adulthood suggesting its function may be pleotropic. In the current study, we focus on changes to the transcriptome of zebrafish larvae in our *prp1^ua5003/ua5003^; prp2^ua5001/ua5001^* mutant fish during early development and identify changes in the transcript abundance of several gene families and related biological processes and further focus on cell adhesion.

Previous transcriptomic and proteomic approaches to investigate changes after the loss of PrP^C^ in mice or mammalian cells have found changes in a consistent set of biological processes including cell adhesion, apoptosis, proteolysis, protection against ROS, the immune system and aspects of the cell cycle [27,33,47,48]. Here, our own transcriptomic approach and comparison of the biological process gene ontologies finds a similar group of processes affected. It is worth noting that we did not see great similarity at the individual gene level with that of other studies. This is likely due to the age of the animals in question. Our zebrafish were 3 days post fertilization (dpf) and would have undergone gastrulation. Similar studies have used either younger zebrafish morphants [49], or E6.7 and E7.5 mice [33] which would not have begun or completed gastrulation. This difference in the relative ages and developmental stages, as well as a different species, may account for this lack of gene expression similarity. We also used whole zebrafish larvae, as opposed to specifically the brain or cell culture, as we were interested in a role of PrP^C^ across the development of the entire organism. There was still a large overlap in the categories of biological process affected overall.

### Prp1 and prp2 regulation of cell adhesion genes during development

One of the more dramatic phenotypes involving prion protein is the gastrulation arrest reported by some scientists in early zebrafish embryos caused by morpholino knockdown of *prp1* leading to disruption of the localization of E-cadherin [18]; however we have been unable to replicate this ourselves (Figure 4). This may be due to a difference in concentration of morpholino. We have previously shown that higher morpholino doses still cause phenotypes in our *prp1* mutant zebrafish, which suggests that the morpholinos have non-specific effects. As such we elected to use lower morpholino doses which did not result in phenotypes in our mutants [17]. As the gastrulation arrest associated with E-cadherin had robust controls demonstrating rescue of the phenotype those results are unlikely to be due to off-target effects at the concentration used [18]. As previous work has also described the effects of PrP^C^ on cell adhesion, particularly the polysialylation of NCAM1 by ST8SIA2 as a requirement for cells to undergo epithelial to mesenchymal transition [21] and through direct interaction with NCAM1 for neuronal differentiation [30], we investigated whether there were similar changes in expression of the zebrafish *ncam1a* and *st8sia2* and further identified significant decreases in transcript abundance of protocadherins. Transcript abundance of *ncam1a* was significantly reduced in our prion mutants, as was the transcript abundance of *st8sia2*.

Cell adhesion is the largest gene ontology category with a significant decrease in transcript abundance in our mutants. There are 38 genes associated with the cell adhesion process affected at the chosen log_2_ fold change cut-off, 31 of which belong to the protocadherin family. Protocadherins are the largest subfamily of cadherin cell adhesion molecules and are primarily expressed within the central nervous system where they are important for its early and continued development [34]. Outside of the chosen fold change cut-off used in the gene ontology analysis are further protocadherins, including members of the *pcdh1* alpha and gamma clusters, and two non-clustered delta protocadherins, *pcdh19* and *pcdh10b*. All of these show a reduction in transcript abundance in our *prp1^ua5003/ua5003^; prp2^ua5001/ua5001^* compound mutants compared to WT.

The age of the zebrafish used for RNA-sequencing was determined by our previous work on *prp1* morphants and *prp2* mutants while trying to capture a time where both genes are expected to be expressed [17,35]. Combined with the *prp1* morpholino data in Figure 4, these results may suggest a role of *prp1* and *prp2* in the expression and regulation of protocadherins and other cell adhesion genes such as *ncam1a* in development of the CNS. Furthermore, genes affected in the FAK pathway would suggest that these processes may be affected through controlling the migration and differentiation of cells which would also support the gastrulation phenotype seen by others [18].

### Prion protein mutant fish show decrease in focal adhesion and actin regulation transcript abundance

Aside from the metabolism KEGG pathway, KEGG analysis shows that the two most affected pathways with a decrease in gene transcript abundance are the focal adhesion kinase pathway and the regulation of actin cytoskeleton pathway. There are 11 genes affected in the FAK pathway and 13 genes affected in the regulation of actin cytoskeleton pathway; between the two there is an overlap of 7 genes. Combined, this suggests the involvement of prion protein in not only cell adhesion processes but also processes which regulate cell motility and differentiation. In addition to cell motility the FAK pathway is heavily involved in angiogenesis [50] which previous transcriptomic studies have shown to be a biological process affected in developing PrP^C^ knockout mice [33].

### Neuroprotection and roles in immune function

Further, of particular interest is the decreased relative transcript abundance of *pcdh19*, a non-clustered protocadherin which has been shown to be one of the highest genetic risk factors relating to epilepsy [51]. Mice lacking PrP^C^ have been shown to be at an increased risk of seizures [52] and we have also shown this in our *prp1^−/-^* and *prp2^−/-^* knockout zebrafish [17,53]. This adds to the increasing amount of data showing PrP^C^ plays a neuroprotective role in vertebrates; this may explain why many phenotypes now becoming apparent occur only after stress is put on the animal.

Finally, Ncam1 has been shown to be expressed in cells involved in the innate immune system including natural killer (NK) cells [54], which also express PrP^C^. The expression of PrP^C^ in immune system cells and tissues is an understudied area of research but there is evidence to suggest it is involved in immune quiescence [55]. This coincides with its higher expression levels in tissues where inflammation could be severely damaging, such as the CNS and testes. Regulation of Ncam1 by PrP^C^ may therefore be a method in which immune suppression is enacted in these tissues to prevent further damage under stress; however more work is required to properly establish this.

### Concluding remarks

To conclude, here we present a transcriptome analysis comparing WT zebrafish and our *prp1^ua5003/ua5003^; prp2^ua5001/ua5001^* mutant zebrafish early in development (3dpf). We find significant changes in transcript abundance of genes in several different biological process gene ontology categories including cell adhesion, proteolysis and oxidation/reduction processes. Importantly, while there is not much overlap at the individual gene level compared to similar studies done in mice our results do overlap considerably at the categorical level. This implies an important, cross-species conserved role of PrP^C^ in the early development of organisms.

The data support past conclusions that PrP^C^ participates in cell adhesion pathways. Further, the data implicate a shared role for PrP^C^ in regulating NCAM1 and its adhesion functions via ST8SIA2; this shared function of PrP^C^ between mammals and fish is consistent with this being part of an ancient role for PrP^C^ early in its evolution [19].

## Materials and methods

### Animal ethics, zebrafish fish lines and husbandry

Zebrafish were raised, maintained and bred following Animal Care and Use Committee: Biosciences procedures at the University of Alberta following guidelines set by the Canadian Council of Animal Care. Fish were kept at the University of Alberta fish facility at 28 ͦC under a 14:10 hour light/dark cycle as previously described [56]. The AB strain of zebrafish was used as WT fish as controls for experiments, as well as the background for the *prp1^ua5003/ua5003^; prp2^ua5001/ua5001^* compound homozygous mutants which we previously generated in our lab [17,23].

### RNA-sequencing analysis of WT and prp1^ua5003/ua5003^; prp2^ua5001/ua5001^ mutant larvae

AB WT and *prp1^ua5003/ua5003^; prp2^ua500/ua5001^* fish (ZFIN ID: ZDB-ALT-181,113-1 and ZDB-ALT-130,724-2) were bred and raised to 3dpf. 50 larvae were taken to form three replicates of each group, WT and mutant, totalling six different samples. Each pool of 50 larvae was homogenized in TRIzol (Invitrogen/ThermoFisher Scientific catalogue no. 15,596,026) with a rotor stator homogenizer (VWR catalogue no. 47,747–370, Radnor, PA) and shipped to Otogenetics (Atlanta, GA) for Illumina PE100-125 and HiSeq2500 sequencing and DNAnexus Platform standard RNAseq analysis at a depth of greater than 41 million reads. Read alignments and annotation were done using the TopHat and Bowtie pipelines and initial quantification analysis of differential gene expression was done using Cufflinks [57,58]. Upon receipt of results it was found that two of the three *prp1^ua5003/ua5003^; prp2^ua5001^*samples might have been contaminated with WT transcripts. We took a conservative approach and filtered these samples out of the analysis. The integrity of the remaining *prp1^ua5003/ua5003^; prp2^ua5001/ua5001^* sample was rigorously screened to ensure it lacked WT transcript by assessing SNPs that were consistently present in mutant vs WT samples. Further analysis was performed using the R Programming Language (Version 4.0.0) packages CummRbund and ggplot2 [59–61].

### RT-qPCR detection of selected genes of interest

Experiments were performed in compliance with the MIQE guidelines (Minimum Information for Publication of Quantitative Real-Time PCR Experiments [62]). RNA samples for all genes were extracted from either 3dpf WT AB or compound homozygous *prp1*^ua5003/ua5003^; *prp2^ua5001/ua5001^* mutant zebrafish. RNA extraction was done from pools of 15–20 larvae previously stored in RNAlater (Ambion/ThermoFisher Scientific, catalogue no. AM7021) and processed using the RNeasy Kit (Qiagen catalogue #74,104, Toronto, ON, Canada) following the manufacturers protocol. Homogenization of larvae was done in RLT buffer with a rotor stator homogenizer as stated above. RNA concentration was quantified using a Nanodrop 2000 spectrophotometer (Thermo Scientific). RNA integrity was confirmed using an Agilent RNA 6000 NanoChip and Agilent 2100 Bioanalyser for numbers of at least 7/10. cDNA was generated using a qScript Supermix kit (Quanta BioSciences catalogue #95,048–100, Beverly, MA, USA) and qPCR carried out as described previously [17]. Three technical replicates were used for each biological replicate and transcript abundance was normalized to β-*actin*. Statistical analysis for relative fold change in transcript abundance was done using RQ values. Primers used for the genes were as follows: *prp1* forward: 5ʹ-ATCCGGCACTTATTGAGCAG-3ʹ, *prp1* reverse: 5ʹ-CACTTCGGAGATGCTGTGTC-3ʹ, *prp2* forward: 5ʹ-CCAACTCTGCAGCTAGTACA-3ʹ, *prp2* reverse: 5ʹ-CAGTGTCGCCGTCATTATCA-3ʹ, *st8sia2* forward: 5ʹ- GACCAACCATGTCCAGATCAAAC-3ʹ, *st8sia2* reverse: 5ʹ- TGGATCTCATCACAAAAGCGAGTA-3ʹ, *ncam1a* forward: 5ʹ-GTAGCTGGAAAAAGGCCCCT-3ʹ, *ncam1a* reverse: 5ʹ-AACAGTGGCAGCTACCTGTC −3ʹ. All primers were validated before use.

For the RT-qPCR primers used for genes related to eye development see supplementary Table 1.

### Morpholino injections in zebrafish embryos

An antisense *prp1* morpholino oligonucleotide (MO) was purchased from Gene Tools, LLC (Philomath, OR) and has been previously described by us and others [18,35] (ZFIN ID: ZDB-MRPHLNO-100,423-6), a standard negative control morpholino was also acquired and used in experiments (5ʹ-CCTCTTACCTCAGTTACAATTTATA-3ʹ). Injection solutions consisted of 1.0 μl KCl, 1.0 μl 0.25% dextran red, MO specific volume resulting in a 5ng/μl concentration for *prp1*-MO or 2.5ng/μl for the standard MO and the volume finalized to 10 μl with nuclease free water. Embryos identified to be at the 1–2 cell stage were mounted on an agarose plate and injected with 1nl of injection solution with the volume previously calibrated using an ocular micrometre, injecting into mineral oil. Larvae at 3dpf were imaged using a Leica M164 dissecting microscope with a Leica DFC 400 camera.

### E-cadherin immunohistochemistry

Embryos identified at the shield stage of development (approximately 6 hpf) were manually dechorionated and fixed in 4% paraformaldehyde and processed for antibody staining. An anti-mouse E-cadherin antibody (BD Biosciences, 610,181) at a 1:5000 dilution was used, and embryos were imaged using a Zeiss LSM 700 scanning confocal microscope and Zen 2010 software (Carl Zeiss Imaging). Images were analysed with ImageJ.

### Gene ontology, KEGG pathway and statistical analysis

Genes identified to have either a log_2_ fold change of 0.5 or greater (increase in transcript abundance) or −0.5 or lower (decrease in transcript abundance) were selected for gene ontology and KEGG pathway analysis using DAVID version 6.8 [63,64]. Additional statistical analysis and visualization was carried out using the tidyverse group of R packages [65] and Microsoft Excel.

## Supplementary Material

Supplemental Material
