# Supplementary material for: Transcriptomic analysis of zebrafish prion protein mutants supports conserved cross-species function of the cellular prion protein
Source: Prion. 2021 Jun 18;15(1):70–81. doi: 10.1080/19336896.2021.1924557 (PMC8216189; doi:10.1080/19336896.2021.1924557)
Supplement: Supplemental Material [file KPRN_A_1924557_SM6152.zip › Supplementary information/FINAL supplementary material.docx]

| Gene name | Forward Primer 5'-3' | Reverse Primer 5'-3' |
| --- | --- | --- |
| *cav1* | TCA ACC GAG ACC CAA AGC AT | CGA AGC TGT AGG TGC CGG |
| *dio1* | GGA TAT CAG CGT GCA CAA AAA C | CAG GGC ATG GAG GGT CTT |
| *otx2* | TCG AAA CTG TGA TCT GTT GTA ACT GTA | AAT CTA TTA AAA TCA CAG CCG AGT CTT |
| *otx5* | ACA GCG GCG CGA AAG A | GGT ATC GGG TTT TGG AGA ACA G |
| *opn1sw2* | CTA TCT TTG CAA TCT GGG TGG TT | AAA GGC AGG AGG GAA TGG TT |
| *opn1sw1* | TCC TCC CGC AGC ACA TTT AC | AAA GTT ACG GGA TTT GAA CAA TCA G |
| *opn1lw2* | CAA GAG CGC CAC CAT CTA CA | ACC TTC TTT CCA AAG AGC TGC |
| *thraa* | CTG AAA GGC TGC TGT ATG GAG AT | TCT CTC CGC TCA GGG TCA GA |
| *cry1aa* | GGC TGC TTG CTT GCA CTA TGT | GGG ACT GAA TAG GTG TAC GAG ACA |
| *sod1* | ACT CTG TCA GGC CAA CAT TCT | ACT TTC CTC ATT GCC ACC CT |
| *crx* | TCT CCT TTA CTT CAG CGG ATT GG | CGC CTC CAC TTG CTG ACA |
| *nr3c1* | AAG CTA CTG GAC TCC ATG CAC | AAA CTC CAC GCT CAG AGA TT |

**Supplementary Table 1:**

**Supplementary Table 1:** RT-qPCR primer list for genes in supplementary figure 1.

**
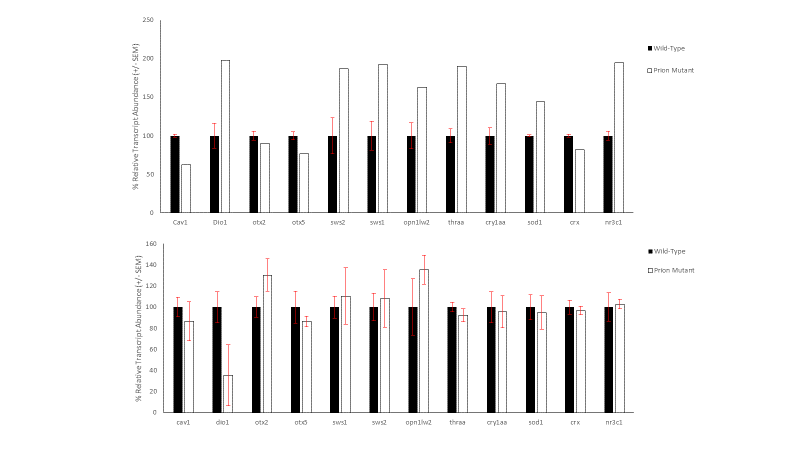
Supplementary Figure 1:**

**Supplementary Figure 1:** Top: RNA-Sequencing of selected genes between wild-type and prion mutant (*prp1^ua5003/ua5003^*;*prp2^ua5001/ua5001^*) homozygous fish. Bottom: Initial RT-qPCR of selected genes associated between wild-type and prion mutant (*prp1^ua5003/ua5003^*;*prp2^ua5001/ua5001^*) homozygous fish. RT-qPCR results did not validate that seen in RNA-sequencing, likely due to their labile abundance over circadian cycles.
