# Supplementary figures and images for: Transcriptomic analysis of zebrafish prion protein mutants supports conserved cross-species function of the cellular prion protein
Source: Prion. 2021 Jun 18;15(1):70–81. doi: 10.1080/19336896.2021.1924557 (PMC8216189; doi:10.1080/19336896.2021.1924557)

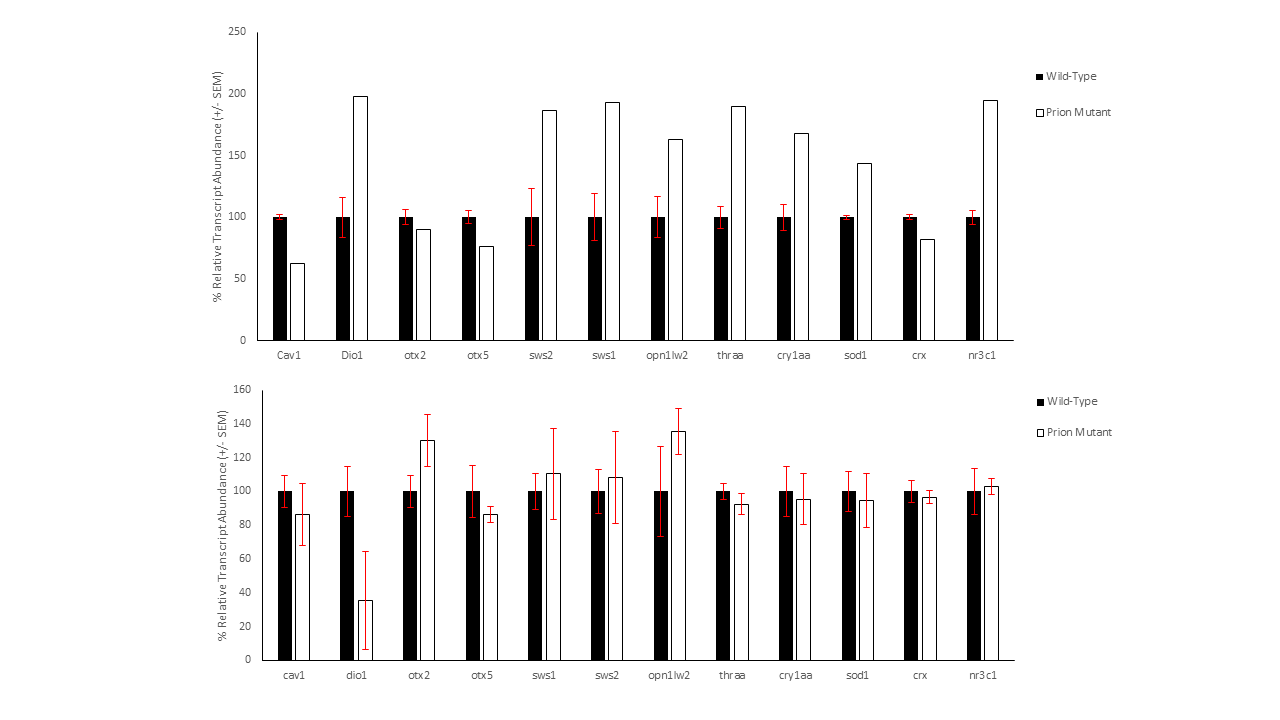

Supplement: Supplemental Material [file KPRN_A_1924557_SM6152.zip › Supplementary information/Supplementary Figure 1.TIF]
